# *S*-Phenyl 4-meth­oxy­benzothio­ate

**DOI:** 10.1107/S1600536812005454

**Published:** 2012-03-17

**Authors:** Adel S. El-Azab, Alaa A.-M. Abdel-Aziz, Hussein I. El-Subbagh, Suchada Chantrapromma, Hoong-Kun Fun

**Affiliations:** a Department of Pharmaceutical Chemistry, College of Pharmacy, King Saud University, PO Box 2457, Riyadh 11451, Saudi Arabia; b Department of Organic Chemistry, Faculty of Pharmacy, Al-Azhar University, Cairo 11884, Egypt; c Department of Medicinal Chemistry, Faculty of Pharmacy, University of Mansoura, Mansoura 35516, Egypt; d Department of Pharmaceutical Chemistry, Faculty of Pharmaceutical Sciences and Pharmaceutical Industries, Future University, Cairo 12311, Egypt; e Crystal Materials Research Unit, Department of Chemistry, Faculty of Science, Prince of Songkla University, Hat-Yai, Songkhla 90112, Thailand; f X-ray Crystallography Unit, School of Physics, Universiti Sains Malaysia, 11800 USM, Penang, Malaysia

## Abstract

In the mol­ecule of the title thio­ester, C_14_H_12_O_2_S, the dihedral angle between the phenyl and benzene rings is 71.8 (3)°. The meth­oxy group is essentially coplanar with the benezene ring to which it is bonded, with an r.m.s. deviation of 0.0065 (5) Å for the non-H atoms involved. In the crystal, weak C—H⋯π inter­actions are present.

## Related literature

For background to and applications of thio­esters, see: Agapiou & Krische (2003 ▶); Choi *et al.* (2003 ▶); El-Azab & Abdel-Aziz (2012 ▶); Horst *et al.* (2007 ▶); Howell *et al.* (2006 ▶); Jew *et al.* (2003 ▶); Liebeskind & Srogl (2000 ▶); McGarvey *et al.* (1986 ▶); Ozaki *et al.* (2003 ▶); Shah *et al.* (2002 ▶); Yang & Drueckhammer (2001 ▶). For related structures and the synthesis of similar compounds, see: Barbero *et al.* (2003 ▶). For bond-length data, see: Allen *et al.* (1987 ▶).
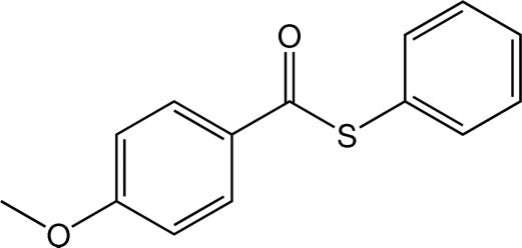


## Experimental

### 

#### Crystal data

C_14_H_12_O_2_S
*M* *_r_* = 244.31
Orthorhombic, 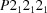

*a* = 5.4478 (2) Å
*b* = 8.2149 (3) Å
*c* = 27.3841 (6) Å
*V* = 1225.52 (7) Å^3^
*Z* = 4
Cu *K*α radiation
μ = 2.23 mm^−1^
*T* = 296 K
0.58 × 0.22 × 0.17 mm

#### Data collection

Bruker SMART APEXII CCD area-detector diffractometer
Absorption correction: multi-scan (*SADABS*; Bruker, 2009 ▶) *T*_min_ = 0.357, *T*_max_ = 0.699
7810 measured reflections
2144 independent reflections
1479 reflections with *I* > 2σ(*I*)
*R*_int_ = 0.050

#### Refinement

*R*[*F* ^2^ > 2σ(*F* ^2^)] = 0.056
*wR*(*F* ^2^) = 0.199
*S* = 1.22
2144 reflections
156 parameters
H-atom parameters constrained
Δρ_max_ = 0.32 e Å^−3^
Δρ_min_ = −0.28 e Å^−3^
Absolute structure: Flack (1983 ▶), 1811 Friedel pairs
Flack parameter: 0.07 (5)

### 

Data collection: *APEX2* (Bruker, 2009 ▶); cell refinement: *SAINT* (Bruker, 2009 ▶); data reduction: *SAINT*; program(s) used to solve structure: *SHELXTL* (Sheldrick, 2008 ▶); program(s) used to refine structure: *SHELXTL*; molecular graphics: *SHELXTL*; software used to prepare material for publication: *SHELXTL* and *PLATON* (Spek, 2009 ▶).

## Supplementary Material

Crystal structure: contains datablock(s) global, I. DOI: 10.1107/S1600536812005454/lh5413sup1.cif

Structure factors: contains datablock(s) I. DOI: 10.1107/S1600536812005454/lh5413Isup2.hkl

Supplementary material file. DOI: 10.1107/S1600536812005454/lh5413Isup3.cml

Additional supplementary materials:  crystallographic information; 3D view; checkCIF report

## Figures and Tables

**Table 1:** Hydrogen-bond geometry (Å, °) *Cg*1 is the centroid of the C1–C6 ring.

*D*—H⋯*A*	*D*—H	H⋯*A*	*D*⋯*A*	*D*—H⋯*A*
C3—H3*A*⋯*Cg*1^i^	0.93	2.96	3.658 (6)	133

Symmetry code: (i) 
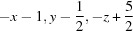
.

**Fig. 1.**
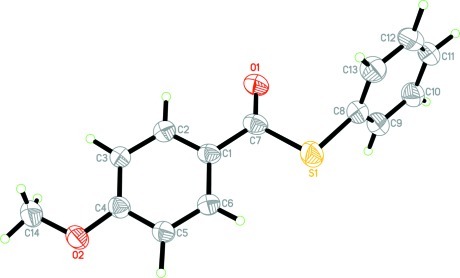
The molecular structure of (I), showing 30% probability displacement ellipsoids and the atom-numbering scheme.

## supplementary crystallographic information

### Comment

Thioesters are one of the most useful building blocks for organic transformations such as in application of C-C coupling for the synthesis of carbonyl compounds in asymmetric aldol reactions. Recently, the α-β-unsaturated thioester analogs have been successfully applied for asymmetric additions which allow the access to chiral intermediates for the synthesis of more complex compounds. Furthermore, they were used in natural product synthesis and also are acting as biologically relevant substances finding application for *in vivo* tumor suppression (Agapiou & Krische (2003); Barbero *et al.*, 2003; Choi *et al.*, 2003; Horst *et al.*, 2007; Howell *et al.*, 2006; Jew *et al.*, 2003; Liebeskind & Srogl 2000; McGarvey *et al.*, 1986; Ozaki *et al.*, 2003; Shah *et al.*, 2002; Yang & Drueckhammer, 2001). Owing to these applications of thioesters, the title compound (I) was synthesized. The molecule is chiral even though it has no chiral center as its mirror image cannot be superposed onto itself. The absolute configuration and crystal structure are reported. We have examined optically the batch of crystals and the morphology is the same for all the crystals in the batch thereby implying that there is no spontaneous resolution.

In the molecule of (I) shown in Fig. 1, the dihedral angle between the phenyl and benzene rings is 71.8 (3)°. The central O1/C7/S1 plane makes dihedral angles of 10.8 (5) and 81.0 (6)° with the C1–C6 and C8–C13 rings, repectively. The methoxy group of the 4-methoxyphenyl group is essentially co-planar with its bound benezene ring with a r.m.s. deviation of 0.0065 (5) Å for the eight non H atoms (C1/C2/C3/C4/C5/C6/O2/C14) and the torsion angle C14—O2—C4—C3 = -2.1 (8)°. The bond distances in (I) are within normal ranges (Allen *et al.*, 1987).

The crystal structure is consolidated by weak C—H···π interactions (Table 1).

### Experimental

The title compound was synthesized according to El-Azab & Abdel-Aziz (2012). The trifluoroacetic acid (0.4 equiv) was added dropwise to a stirred solution of carboxylic acid (1 equiv) and thiophenol (1 equiv) in dry CH_3_CN (0.01 mol/l) over a period of 15 min at room temperature. After being stirred for 2–5 h at 333 K, the mixture was quenched by adding ammonium chloride solution (5 ml), extracted with ethylacetate, washed with brine and dried over anhydrous sodium sulfate. The product obtained after the evaporation of the solvent was purified by colum chromatography using mixture of hexane and CHCl_3_ as eluent. The crystal was obtained by slow evaporation of the eluent system hexane and CHCl_3_; m.p. 366-367 K, 97% yield. IR (KBr): 1661 cm^-1^ (CO), ^1^H NMR (CDCl_3_): d 8.06 (d, 2H, J = 8.5 Hz), 7.55–7.54 (m, 2H), 7.48 (m, 3H), 6.99 (t, 2H, J = 4.0 Hz), 2.90 (s, 3H). ^13^C NMR (CDCl_3_): d 55.6, 113.9, 127.7, 129.2, 129.4, 129.8, 135.2, 164.0, 188.6.

### Refinement

All H atoms were placed in calculated positions with d(C—H) = 0.93 for aromatic and 0.96 Å for CH_3_ atoms. The *U*_iso_ values were constrained to be 1.5*U*_eq_ of the carrier atom for methyl H atoms and 1.2*U*_eq_ for the remaining H atoms. A rotating group model was used for the methyl groups. 1811 Friedel pairs were used to determine the absolute configuration.

### Crystal data

**Table:**

C_14_H_12_O_2_S	*D*_x_ = 1.324 Mg m^−^^3^
*M**_r_* = 244.31	Melting point = 366–367 K
Orthorhombic, *P*2_1_2_1_2_1_	Cu *K*α radiation, λ = 1.54178 Å
Hall symbol: P 2ac 2ab	Cell parameters from 2144 reflections
*a* = 5.4478 (2) Å	θ = 3.2–69.4°
*b* = 8.2149 (3) Å	µ = 2.23 mm^−^^1^
*c* = 27.3841 (6) Å	*T* = 296 K
*V* = 1225.52 (7) Å^3^	Needle, colourless
*Z* = 4	0.58 × 0.22 × 0.17 mm
*F*(000) = 512	

### Data collection

**Table:**

Bruker SMART APEXII CCD area-detector diffractometer	2144 independent reflections
Radiation source: fine-focus sealed tube	1479 reflections with *I* > 2σ(*I*)
Graphite monochromator	*R*_int_ = 0.050
φ and ω scans	θ_max_ = 69.4°, θ_min_ = 3.2°
Absorption correction: multi-scan (*SADABS*; Bruker, 2009)	*h* = −4→6
*T*_min_ = 0.357, *T*_max_ = 0.699	*k* = −9→8
7810 measured reflections	*l* = −32→29

### Refinement

**Table:**

Refinement on *F*^2^	Hydrogen site location: inferred from neighbouring sites
Least-squares matrix: full	H-atom parameters constrained
*R*[*F*^2^ > 2σ(*F*^2^)] = 0.056	*w* = 1/[σ^2^(*F*_o_^2^) + (0.0897*P*)^2^ + 0.2372*P*] where *P* = (*F*_o_^2^ + 2*F*_c_^2^)/3
*wR*(*F*^2^) = 0.199	(Δ/σ)_max_ = 0.001
*S* = 1.22	Δρ_max_ = 0.32 e Å^−^^3^
2144 reflections	Δρ_min_ = −0.28 e Å^−^^3^
156 parameters	Extinction correction: *SHELXTL* (Sheldrick, 2008), Fc^*^=kFc[1+0.001xFc^2^λ^3^/sin(2θ)]^-1/4^
0 restraints	Extinction coefficient: 0.025 (3)
Primary atom site location: structure-invariant direct methods	Absolute structure: Flack (1983), with 1811 Friedel pairs
Secondary atom site location: difference Fourier map	Flack parameter: 0.07 (5)

### Special details

**Table:**

Geometry. All esds (except the esd in the dihedral angle between two l.s. planes) are estimated using the full covariance matrix. The cell esds are taken into account individually in the estimation of esds in distances, angles and torsion angles; correlations between esds in cell parameters are only used when they are defined by crystal symmetry. An approximate (isotropic) treatment of cell esds is used for estimating esds involving l.s. planes.
Refinement. Refinement of F^2^ against ALL reflections. The weighted R-factor wR and goodness of fit S are based on F^2^, conventional R-factors R are based on F, with F set to zero for negative F^2^. The threshold expression of F^2^ > 2sigma(F^2^) is used only for calculating R-factors(gt) etc. and is not relevant to the choice of reflections for refinement. R-factors based on F^2^ are statistically about twice as large as those based on F, and R- factors based on ALL data will be even larger.

### Fractional atomic coordinates and isotropic or equivalent isotropic displacement parameters (Å^2^)

**Table:**

	*x*	*y*	*z*	*U*_iso_*/*U*_eq_	
S1	0.2447 (3)	0.2241 (2)	0.88540 (4)	0.0866 (6)	
O1	−0.1014 (8)	0.0079 (5)	0.87109 (10)	0.0848 (13)	
O2	−0.0150 (7)	−0.0916 (5)	1.10026 (10)	0.0712 (10)	
C1	0.0101 (8)	0.0386 (6)	0.95439 (14)	0.0579 (11)	
C2	−0.1770 (8)	−0.0607 (6)	0.97065 (16)	0.0641 (12)	
H2A	−0.2939	−0.0981	0.9486	0.077*	
C3	−0.1940 (8)	−0.1055 (7)	1.01918 (14)	0.0657 (13)	
H3A	−0.3225	−0.1713	1.0297	0.079*	
C4	−0.0204 (8)	−0.0527 (6)	1.05180 (14)	0.0596 (11)	
C5	0.1682 (8)	0.0475 (6)	1.03636 (15)	0.0637 (12)	
H5A	0.2841	0.0853	1.0585	0.076*	
C6	0.1830 (8)	0.0911 (7)	0.98763 (15)	0.0625 (12)	
H6A	0.3117	0.1568	0.9771	0.075*	
C7	0.0229 (9)	0.0742 (7)	0.90125 (15)	0.0653 (13)	
C8	0.2346 (9)	0.2180 (7)	0.82059 (16)	0.0684 (13)	
C9	0.4109 (10)	0.1329 (7)	0.79629 (16)	0.0773 (15)	
H9A	0.5299	0.0757	0.8136	0.093*	
C10	0.4122 (11)	0.1319 (8)	0.74530 (17)	0.0827 (17)	
H10A	0.5297	0.0722	0.7284	0.099*	
C11	0.2390 (10)	0.2194 (7)	0.72047 (17)	0.0779 (14)	
H11A	0.2402	0.2198	0.6865	0.094*	
C12	0.0654 (11)	0.3056 (9)	0.74480 (18)	0.0860 (18)	
H12A	−0.0511	0.3651	0.7275	0.103*	
C13	0.0619 (11)	0.3049 (8)	0.79550 (19)	0.0818 (16)	
H13A	−0.0574	0.3634	0.8123	0.098*	
C14	−0.2089 (11)	−0.1900 (9)	1.11832 (18)	0.096 (2)	
H14A	−0.1840	−0.2103	1.1525	0.144*	
H14B	−0.2115	−0.2915	1.1010	0.144*	
H14C	−0.3624	−0.1347	1.1137	0.144*	

### Atomic displacement parameters (Å^2^)

**Table:**

	*U*^11^	*U*^22^	*U*^33^	*U*^12^	*U*^13^	*U*^23^
S1	0.1088 (11)	0.0977 (13)	0.0532 (6)	−0.0329 (9)	−0.0016 (6)	−0.0022 (6)
O1	0.088 (3)	0.107 (4)	0.0589 (17)	−0.019 (2)	−0.0185 (16)	0.0018 (19)
O2	0.083 (2)	0.079 (3)	0.0521 (16)	−0.0038 (17)	−0.0032 (13)	0.0042 (16)
C1	0.058 (2)	0.064 (3)	0.052 (2)	0.0023 (19)	−0.0063 (17)	−0.011 (2)
C2	0.061 (3)	0.066 (4)	0.065 (2)	−0.006 (2)	−0.0035 (18)	0.001 (2)
C3	0.061 (3)	0.083 (4)	0.053 (2)	−0.013 (2)	−0.0014 (17)	0.003 (2)
C4	0.066 (3)	0.060 (3)	0.053 (2)	−0.001 (2)	−0.0001 (17)	−0.006 (2)
C5	0.066 (3)	0.069 (4)	0.056 (2)	−0.008 (2)	−0.0074 (17)	0.000 (2)
C6	0.061 (3)	0.066 (4)	0.060 (2)	−0.009 (2)	−0.0048 (18)	−0.001 (2)
C7	0.066 (3)	0.077 (4)	0.053 (2)	0.008 (2)	−0.0078 (18)	−0.012 (2)
C8	0.069 (3)	0.077 (4)	0.058 (2)	−0.008 (3)	−0.0016 (19)	0.005 (2)
C9	0.077 (3)	0.087 (4)	0.068 (3)	0.001 (3)	−0.003 (2)	0.013 (3)
C10	0.080 (3)	0.102 (5)	0.067 (3)	0.004 (3)	0.007 (2)	0.004 (3)
C11	0.085 (3)	0.096 (4)	0.053 (2)	−0.005 (3)	−0.005 (2)	0.011 (2)
C12	0.081 (4)	0.108 (5)	0.069 (3)	0.006 (3)	−0.015 (3)	0.013 (3)
C13	0.080 (3)	0.086 (5)	0.080 (3)	0.007 (3)	0.002 (2)	0.000 (3)
C14	0.103 (4)	0.120 (6)	0.065 (3)	−0.033 (4)	0.004 (3)	0.014 (3)

### Geometric parameters (Å, º)

**Table:**

S1—C8	1.776 (4)	C6—H6A	0.9300
S1—C7	1.779 (6)	C8—C9	1.362 (7)
O1—C7	1.199 (5)	C8—C13	1.366 (7)
O2—C4	1.366 (5)	C9—C10	1.397 (6)
O2—C14	1.419 (6)	C9—H9A	0.9300
C1—C6	1.379 (6)	C10—C11	1.367 (7)
C1—C2	1.380 (6)	C10—H10A	0.9300
C1—C7	1.486 (6)	C11—C12	1.356 (8)
C2—C3	1.382 (6)	C11—H11A	0.9300
C2—H2A	0.9300	C12—C13	1.389 (7)
C3—C4	1.371 (6)	C12—H12A	0.9300
C3—H3A	0.9300	C13—H13A	0.9300
C4—C5	1.383 (6)	C14—H14A	0.9600
C5—C6	1.384 (6)	C14—H14B	0.9600
C5—H5A	0.9300	C14—H14C	0.9600
			
C8—S1—C7	101.7 (2)	C9—C8—S1	118.7 (4)
C4—O2—C14	117.1 (4)	C13—C8—S1	120.6 (4)
C6—C1—C2	118.5 (4)	C8—C9—C10	119.7 (5)
C6—C1—C7	123.6 (4)	C8—C9—H9A	120.2
C2—C1—C7	117.8 (4)	C10—C9—H9A	120.2
C1—C2—C3	121.1 (4)	C11—C10—C9	119.4 (5)
C1—C2—H2A	119.4	C11—C10—H10A	120.3
C3—C2—H2A	119.4	C9—C10—H10A	120.3
C4—C3—C2	119.7 (4)	C12—C11—C10	120.7 (4)
C4—C3—H3A	120.1	C12—C11—H11A	119.6
C2—C3—H3A	120.1	C10—C11—H11A	119.6
O2—C4—C3	125.0 (4)	C11—C12—C13	119.9 (5)
O2—C4—C5	114.9 (4)	C11—C12—H12A	120.0
C3—C4—C5	120.1 (4)	C13—C12—H12A	120.0
C4—C5—C6	119.5 (4)	C8—C13—C12	119.7 (5)
C4—C5—H5A	120.3	C8—C13—H13A	120.2
C6—C5—H5A	120.3	C12—C13—H13A	120.2
C1—C6—C5	121.0 (4)	O2—C14—H14A	109.5
C1—C6—H6A	119.5	O2—C14—H14B	109.5
C5—C6—H6A	119.5	H14A—C14—H14B	109.5
O1—C7—C1	124.0 (5)	O2—C14—H14C	109.5
O1—C7—S1	122.0 (4)	H14A—C14—H14C	109.5
C1—C7—S1	114.0 (3)	H14B—C14—H14C	109.5
C9—C8—C13	120.5 (5)		
			
C6—C1—C2—C3	0.8 (7)	C6—C1—C7—S1	−12.0 (6)
C7—C1—C2—C3	176.9 (5)	C2—C1—C7—S1	172.1 (3)
C1—C2—C3—C4	−0.9 (8)	C8—S1—C7—O1	−5.6 (5)
C14—O2—C4—C3	−2.2 (8)	C8—S1—C7—C1	173.6 (4)
C14—O2—C4—C5	178.1 (5)	C7—S1—C8—C9	−99.8 (5)
C2—C3—C4—O2	−178.6 (5)	C7—S1—C8—C13	84.1 (5)
C2—C3—C4—C5	1.2 (8)	C13—C8—C9—C10	−1.3 (8)
O2—C4—C5—C6	178.4 (5)	S1—C8—C9—C10	−177.4 (5)
C3—C4—C5—C6	−1.3 (8)	C8—C9—C10—C11	1.4 (9)
C2—C1—C6—C5	−0.9 (8)	C9—C10—C11—C12	−0.6 (9)
C7—C1—C6—C5	−176.8 (5)	C10—C11—C12—C13	−0.3 (9)
C4—C5—C6—C1	1.2 (7)	C9—C8—C13—C12	0.4 (9)
C6—C1—C7—O1	167.1 (5)	S1—C8—C13—C12	176.5 (5)
C2—C1—C7—O1	−8.8 (8)	C11—C12—C13—C8	0.4 (10)

### Hydrogen-bond geometry (Å, º)

*Cg*1 is the centroid of the C1–C6 ring.

**Table:**

*D*—H···*A*	*D*—H	H···*A*	*D*···*A*	*D*—H···*A*
C3—H3*A*···*Cg*1^i^	0.93	2.96	3.658 (6)	133

Symmetry code: (i) −*x*−1, *y*−1/2, −*z*+5/2.
